# Mental health: an Indian perspective

**DOI:** 10.1186/1753-6561-7-S5-O19

**Published:** 2013-08-30

**Authors:** KA Kumar

**Affiliations:** 1 Kerala Institute of Medical Sciences, Trivandrum, India

## 

In the Indian scenario, research in mental health is notably deficient, but available clinical and epidemiological data suggest a significant co-morbidity between mental diseases and cardiovascular diseases. Mental health problems do not have the precision of other biological sciences due to the complex phenotypes. In India the effects of culture and the transition in symptomatology of psychiatric patients are also important. Equally important are the family influences, traditional Indian herbal ethno-pharmacology and the community care perspective. The Indian systems of medicine give a lot of attention to visceral functioning and psychiatric research look into the metabolic substrate.

